# 2507. Sex and race disparities in hepatocellular carcinoma surveillance in patients with chronic hepatitis B: a single center retrospective review

**DOI:** 10.1093/ofid/ofad500.2125

**Published:** 2023-11-27

**Authors:** William Reiche, Stephen Cooper, Christopher Destache, Suhail Sidhu, Bryce Schutte, Darby Keirns, Elezabeth mac, Ian Ng, Haitam Buaisha, Manasa Velagapudi

**Affiliations:** CHI Creighton University Medical Center, Omaha, NE, USA, Omaha, Nebraska; CHI Creighton University Medical Center, Omaha, NE, USA, Omaha, Nebraska; Creighton University School of Pharmacy, Omaha, Nebraska; Creighton University School of Medicine, Omaha, Nebraska; CHI Creighton University Medical Center, Omaha, NE, USA, Omaha, Nebraska; Creighton University School of Medicine, Omaha, Nebraska; CommonSpirit Health Specialty Pharmacy, Phoenix, AZ, USA, Phoenix, Arizona; CHI Creighton University Medical Center, Omaha, NE, USA, Omaha, Nebraska; CHI Creighton University Medical Center, Omaha, NE, USA, Omaha, Nebraska; CHI Health - Creighton University Medical Center - Bergan Mercy, Omaha, Nebraska

## Abstract

**Background:**

The management of patients with chronic hepatitis B (CHB) is complex and involves interdisciplinary care across multiple medical specialties. As a result of this complexity, patients with CHB often do not receive adequate monitoring including HCC surveillance with abdominal ultrasonography (AUS). Previous studies have identified multiple factors associated with decreased HCC surveillance. We aimed to study race and sex disparities in HCC surveillance in patients with CHB during the COVID-19 pandemic.

**Methods:**

We performed a single center retrospective cohort study of patients treated for CHB between January 2018 and January 2022 in Gastroenterology and Infectious Diseases clinics at our institution. HCC surveillance trends for both sexes were further stratified by 6-month time intervals throughout the study period to compare surveillance during the COVID-19 surge at our region and after the surge. The COVID-19 pandemic surge was defined as time 1/1/2020-6/30/2021. Differences between sex and race were evaluated using the chi-square test, Fisher's exact test, and continuous variables were analyzed using analysis of variance (ANOVA).

**Figure:**
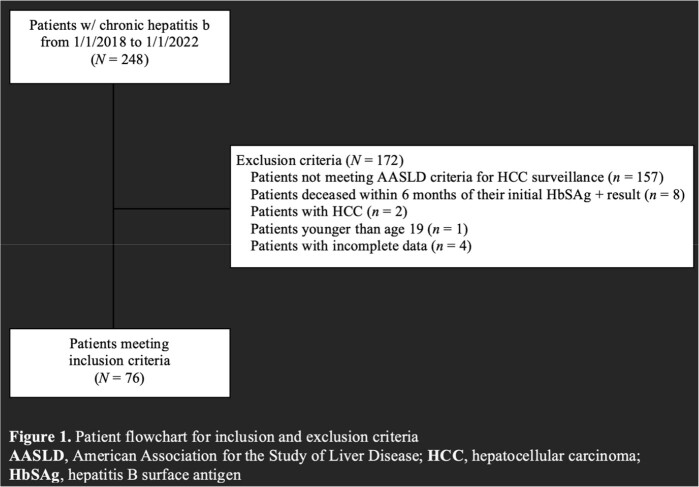

**Results:**

A total of 223 adult patient records between January 2018 and January 2022 were evaluated. In total 37% females were adequately screened for HCC in any of the 6-month time frames compared to 26% for males, p >0.05. During the COVID-19 surge, surveillance rates were reduced in both males and females; however, men had significantly reduced HCC screening compared to women (men 38% compared to women 47%, *P* = 0.011, Figure 1). When comparing COVID-19 HCC screening, stratified by race, 20% Asians, 34% African Americans, and 22% Caucasians were screened by AUS, *P* = 0.069(Figure 2).

Patient Demographics

**Figure:**
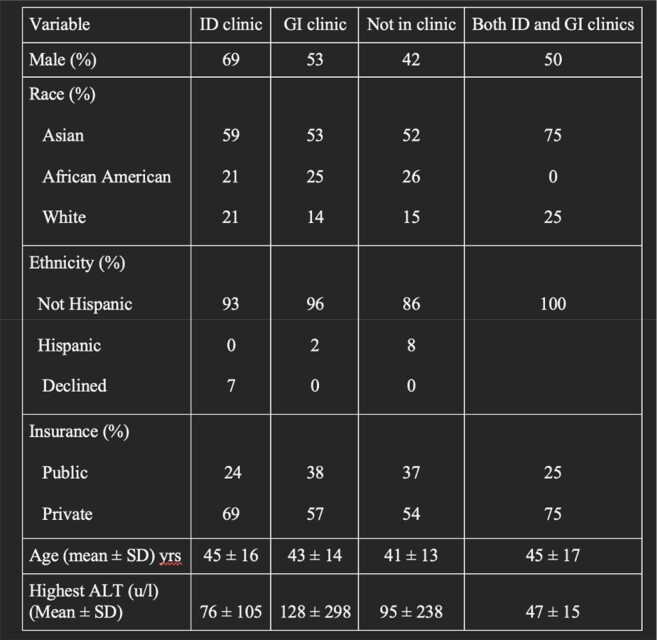

ID, infectious diseases clinic; GI, gastroenterology clinic; SD, standard deviation

**Figure:**
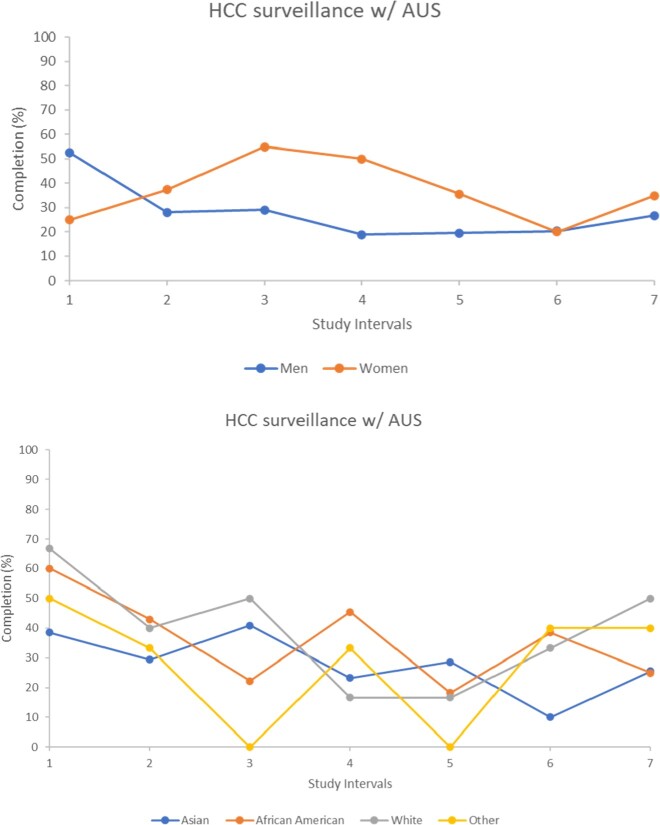

**Conclusion:**

Males received significantly less HCC surveillance compared to females. These differences continued during the COVID-19 pandemic surge. Obtaining appropriate surveillance is important and identifying disparities enables treating physicians to work on measures to mitigate them.

**Disclosures:**

**All Authors**: No reported disclosures

